# Address to the Graduates at the Eleventh Annual Commencement of Niagara Uuiversity Medical College1Delivered at the Academy of Music, Buffalo, May 9, 1894.

**Published:** 1894-08

**Authors:** Harlow C. Curtiss

**Affiliations:** Professor of Medical Jurisprudence


Buffalo Medical and Surgical Journal

Vol. XXXIV. AUGUST, 1894. No. 1.

### Original Address.

### ADDRESS TO THE GRADUATES AT THE ELEVENTH ANNUAL COMMENCEMENT OF NIAGARA UNIVERSITY MEDICAL COLLEGE.[1]

By HARLOW C. CURTISS, A. M.,
Professor of Medical Jurisprudence.

We have just completed the Columbian year with all its pomp and ceremony; with all the eloquence of the gifted speakers of America; with all the wonderful creations of American inventive genius; with the triumphs of our mechanics, engineers and electricians, we have emphasized the rapid progress made in the development of skilled and scientific labor, in which we lead the world As by a magician's wand, there arose on the shores of our inland ocean, suddenly, as in a night, that marvelous White City, whose entrancing beauty—apparently not of this world, but in reality a grand, a concentrated expression of the artistic accomplishments of American landscape engineering, the architect's pencil, the sculptor's chisel—dazzled the wondering eyes of the men of all nations, who hurried thither from every quarter of the globe to exhibit in those magic storehouses the fruits of their industry, their creations of art, their achievements in science.

Truly, there at Chicago the nations vied with each other to show to the world, each for itself, what its own people had accomplished in the strife to push ever onward the steady march of Human Progress. It was as if all the world had taken for its motto the words "*Religio, Cultura, Mores*." How much mankind has accomplished under the inspiration of these three ideals: How much have we failed: How much more must we strive in the future. But, suddenly as a dream, the dazzling, beautiful

1. Delivered at the Academy of Music, Buffalo, May 9, 1894.

White City has vanished; the men of all nations have scattered themselves throughout the world; they still must continue to struggle on in the round of daily tasks, all through the unnumbered cycles of time, until at last "the trump shall sound," and then, as we were in this our Columbian year, dazzled by the magic beauty of our White City by the Lake, then to our almost blinded sight may be unfolded the glories of the beatific vision, then we shall fully realize how feeble have been our best efforts, how small their results. But we shall rejoice if we have done our humble best in a lifelong struggle to perfect ourselves in "*Religio, Cultura, Mores.*" May it then be granted to each one of us to know that the world is not any worse for our having lived in it.

During this our Columbian year we have given much of our attention to the times in which our great discoverer lived, the conditions that moulded him in his development, the motives and influences that impelled him to continue in his daring advance onward through the world of unknown waters to the land of his dreams. Now, let us, as men interested in humanity, in medicine, the healing art, consider the conditions which necessitated the establishment of this our benign mother, the Medical Department of Niagara University, the progress that has been made by her, and the motives which inspire the carrying on of her work.

Niagara University was founded by the priests of the congregation of the mission in the year 1856, and in the year 1863 was incorporated by the legislature of the State of New York under the name of the Seminary of Our Lady of Angels. It was erected in 1883 into a university, under its present title, by the regents of the University of the State of New York, with the full powers and authority of a university. The college of arts of the university is situated on the highest point of the Monteagle Ridge, at Niagara Falls, a location unsurpassed in beauty and grandeur; beautiful views are seen at various points of the college grounds; from the great waterfall the course of the river can be followed over the wild rapids tumbling through the narrow green-clad gorge, on and on until we see in the distance the deep blue of Lake Ontario. Here, immersed in the rest and quiet of the virgin forest, with the beauties of Nature spread before them by a bountiful Creator, the men of Niagara cannot fail to draw in with each breath deep draughts from the fountain of knowledge established by the reverend fathers and prove themselves faithful to the beautiful motto of the university, *Religio, Mores, Cultura.*

In the year 1883, the authorities of the University, realizing the needs of the great city at the upper end of their beautiful Niagara, and feeling the great necessity of a complete and more thorough education for the medical men of their vicinage than was then afforded by the various medical faculties of the day, determined to establish in Buffalo a school of medicine, which should afford a complete and thorough education in that science and art to cover a course of at least three years, instead of a course of but two years, the usual length of the course prescribed by most of the medical faculties in the United States at that time. So, calling to their assistance many of the leading medical men of Buffalo and the vicinity, men unsurpassed by any in the depth of their learning, the extent of their accomplishments, the proficiency and skill of their practice, the wideness and honor of their reputations, they founded in that year the Medical Department of Niagara University.

The medical faculty at once established a course in medicine covering a period of three years, and has ever since carried on the work of the medical school with great thoroughness. So much so, that now, at the end of some eleven years from the inception of the school, the Niagara graduates in medicine are everywhere found in the front rank of their profession. The wisdom of the founders of the medical school in insisting upon the three years' course, has been most forcibly demonstrated by the fact that the University of the State of New York has only recently insisted upon a three years' course being followed by the candidates for the degree of Doctor of Medicine in all the medical schools of the state. [This, in obedience to a law that the Medical Society of the State of New York strove, for ten years, to establish.—Editor.] It is the desire of every member of the medical faculty of Niagara University (unless I am greatly in error) that the course in medicine shall be further lengthened to four years. And this, I doubt not, will be done at some convenient time in the near future.

Here, in America, we have presented to us special needs and conditions which are so broad and widespread in their ramifications that they concern all classes of society, and influence and affect the relations and accomplishments of the men of every calling in the community. The absence of an hereditary superior class, the necessity of building from the foundations in the whole social fabric, the prevalence of intelligence and education, the restlessness of ambition and the strife for betterment, the sense of personal worth, contempt of tradition and conventionalities ; self reliance, the adaptability to circumstances and the ability to conform circumstances to desires ; the stimulating climate, fertility of the soil ; the richness of the rewards secured by effort in every department of human labor, the regard for learning, the prevalent tone of religious thought, the pride of citizenship, the atmosphere of freedom, civic pride ; the mingling of races and ideas ; these, among other influences, have contributed largely to the development in the United States of a special and peculiar type of manhood ; to a complex condition of the social community ; to special and peculiar influences and needs, which we have in common with no other people. Among such people, there being always a close relationship between the character of the medical advisers of a people and the character of the people themselves, it is inevitable that there should be a special type of medical man ; that our system of medicine and the laws governing it should materially differ from those prevalent elsewhere in the civilized world. Here the dominant ideas of liberty (often degenerating down to the level of unrestrained license) have had a direct and powerful influence upon our system of medicine.

In many of our states the practice of medicine is still thrown open to any man who chooses to hold himself out as a "Doctor," without any special license to practise, without any special qualifications ; and the confiding public is forced to choose and sift out for itself, often at the expense of the public health, the good from the bad, the quack and the charlatan from the medical man of sound professional skill and attainments. The popular theory is that each individual is free to choose for himself his own system of medicine as well as his own religion. Governmental paternalism, which tries to protect each individual from the hollow and false, has no tolerance in the minds of our liberty-loving people. Individualism in medicine, as in everything else, has full swing, and the needs of the social community are not considered at all in comparison with the right of the individual to choose for himself. There is no vagary so wild, and no pretension so preposterous, but that it shall receive a respectful hearing with our American people. But although with us a greater proportionate number of individuals have devoted themselves to the art of healing than among the people of any other nation, the good sense of our people has constantly been effective in sifting out the good from the bad, sterling worth from its imitation, reality from hollow pretense, and the standard of professional attainments, required by public opinion, of medical practitioners, has steadily advanced, both in general culture and in technical knowledge. Also, of late there has steadily grown up a feeling that the commonwealth should give to each individual member of the community the protection of governmental guaranty that every person who claims to be a healer of disease, is really qualified as such; particularly is this so in the State of New York, where a state license to practise is now required from every physician, and where state examinations at the end of a three years' course of study in a school of medicine are required before the license is granted.

The demands of the present day as to the training of him who would practise the art of healing are multiplying greatly; the interests at stake are too great to allow the neglect of any possible means of useful knowledge in the various branches of science and learning. The complexity, abstruseness and widespreading relations of the various branches of knowledge necessary to a well-learned medical man are such, that only well-trained minds should essay the study of medicine. A full course of training in such preliminary studies as tend to develop the reasoning faculties and powers of observation, and to equip the mind with a store of general knowledge, is now required by the intelligent opinion of our American public of each would-be medical man before he begins the study of his profession; nay, I might say further, that experience demands it. The ability to master the principles and to achieve skill in the practice of the medicine of today requires in the highest degree a trained eye, a trained mind and a trained hand. Each year witnesses in this country a marked increase in the facilities for obtaining this necessary training; no men have been more ready to realize its importance, and to impress on the general public its necessity, than the great body of medical men acting as individuals, and by means of their organized medical societies. The advance in medical education has been steadily going on, and is still in progress.

Niagara has always led in this advance, and she will never be found backward in the effort to place her graduates in the very foreground of medical progress and enlightenment. She has ever before her the principles set forth in the motto *Religio, Mores, Cultura.* Her students are required to bring with them at the very outset minds already well disciplined in the study of the humanities and the sciences. She has already multiplied, systematized and lengthened the branches of her medical course. She has well-equipped laboratories and a dispensary ; and the well-furnished hospitals which are under the care of the members of her faculty afford to her students opportunities for clinical training unexceled in the United States ; so that, as the only non-medical member of her medical faculty, the speaker can with propriety say that within her doors can be had a practical and theoretical training such as should place her graduates at the forefront of the medical profession. The friends of the college will not, however, simply be pleased with what has been done in the past, but they will ever strive to develop her resources ; to accelerate her progress ; to widen her influences ; she will always strive to furnish her students the best and most comprehensive training ; a high ideal of what a medical man should be ; a keen appreciation of the value of positive knowledge and trained skill ; a steady elevation to the highest types of training and attainment.

The student at Niagara, if he graduates, graduates a well-trained and accomplished physician, worthy to wear the hood of the doctor of medicine, and to assume the awful responsibility of the care of human life. In the days of old (as is now the case in many of the universities of Europe) it was required of him who aspired to wear the hood of the doctor, either in law, theology or medicine, that he should enter the lists of controversy with other doctors, and prove himself worthy of the hood before he could receive that honor. Those who this evening are to receive from the hands of our Rt. Rev. Chancellor the hood of the doctor of medicine are champions in a like contest ; they have heard and answered successfully the questions of many doctors, who have tested their knowledge and certified that they are worthy to wear the hood of red and blue—red, the color of life, of love and of charity ; and blue, the color of truth and mercy.

Gentlemen of the graduating class, we gladly greet you by your new titles of doctors of medicine. May your lives always be distinguished by love and charity, by truth and mercy, as your garb is marked by the red and the blue of your doctors' hood. During the three long years of your course in medicine you have been courageous, determined and diligent. You have laid well the foundations of knowledge upon which you must strive to build all the days of your life. Never forget that medicine is a progressive science and that you can keep up with the progress and advancement of medical science only by constant, hard study, for medicine, like the law, is a jealous mistress.

The days of your education as pupils under instruction are now over. You are now fresh from the class room and the laboratory, and your diplomas today seem very broad with your list of accomplishments. They will begin to shrink from this very hour. Your acquaintance with the various branches of your studies is much greater than it will be in a year from now, and it will probably be still less two years hence, and when you shall be gray-haired practitioners, the progress of medical knowledge will probably have far outrun the various text-books of your college course. "Science," says Doctor Holmes, "is a great traveler, and wears her shoes out very fast," as might be expected. But do not infer that your teachers were in fault when they furnished you with mental stores, likely in a few years to lose their place in your memory. There are many things which we can afford to forget, which it was well to learn. Your mental condition is not the same as if you had never known what you now try in vain to recall. There is a perpetual metempsychosis of thought, and the knowledge of today finds a soil in the forgotten facts of yesterday. When you come to handle life and death as your daily business, your memory will of itself bid good-bye to such knowledge as is not wanted. Your education has, however, been very largely a practical one ; medicine and surgery you have not only studied in books, but at the bedside and the operating table.

It has been your special advantage that you have been afforded the opportunity of seeing much disease in your three years here, and of seeing many cases of the same kind of disease brought together ; for it must be confessed that the great hospitals, infirmaries and dispensaries of a great city like ours are the true centers of medical education. Your minds are now stored with a vast amount of skill and wisdom which you have learned from your various medical instructors, and you would probably know just what to do to meet any one of the many emergencies which confront the physician and the surgeon. You could probably pass a better medical examination today than any one of your instructors; and, however humble-minded you are, time and experience only can teach you the limitations of your knowledge. For the knowledge that you now possess will be a different thing after long habit has made it a part of yourself. Experience means the knowledge gained by long and constant trial, and "an expert," it has been well said, "is one who has been in the habit of trying." "Book-knowledge, lecture-knowledge, examination-knowledge," says Doctor Holmes, "are all in the brain; but work knowledge is not only in the brain, it is in the senses, in the muscles, in the ganglia of the sympathetic nerves. The young man knows the rules, but the old man knows the exceptions; the young man knows his patient, but the old man knows also his patient's family, dead and alive, up and down for generations." The old practitioner has the experience, while the young man has drawn his learning from the latest existing condition of knowledge, and he keeps step with the march of progress much more easily than his seniors.

Do you, gentlemen, in the spare moments of your early years of practice, and, in fact, throughout your whole professional life, read and study and familiarize yourselves with all the latest achievements of medical science and experience will, of itself, come to you; so that if you are true to yourselves, you will go on improving as medical men with each year of your lives. You are now about to enter into relations with the public; to expend your knowledge and skill for its benefit, and to derive your support from the rewards of your labor. You must, first of all, get the confidence of the community, and there is but one way of doing this, that is, *deserve* it. But to deserve it in full measure, you must unite in yourself many excellencies, natural and acquired; you cannot do better than begin by conforming to the standard of the ancient oath of Hippocrates. Enter your patient's house with the sole purpose of doing him good; and so conduct yourselves as to avoid the very appearance of evil. Never betray confidence reposed in you; avoid all that is mean and low; shun narrowmindedness. Have the genuine good manners that arise from cheerfulness of disposition and cleanness of life. The three graces of manners are gentleness, cheerfulness and urbanity. Cultivate them. Good manners are a part of good morals, and more failures in life have been caused by defects in morals than by defects in intellect. But, above all, conduct yourselves as gentlemen. "What is it," asks Thackery, "to be a gentleman? Is it to have lofty aims, to lead a pure life, to keep your honor virgin, to have the esteem of your fellow-citizens and the love of your fireside, to bear good fortune meekly, to suffer evil with constancy, and through evil or good to maintain truth always? Show me the happy man whose life exhibits these qualities, and him we will salute as gentleman, whatever his rank may be." If your lives, gentlemen, exhibit those qualities, you will need no code of medical ethics.

Endeavor to realize always your great responsibilities; and, following the steps of the Great Physician, let your lives bestow upon those who are in need, love, charity, kindness, mercy, truth. Whether you have succeeded or failed, time will indicate, but eternity will decide.

Gentlemen of the graduating class, the intercourse of teacher and student between us all is now ended. We bid you welcome as medical practitioners; the world lies open before you; old Niagara will soon be to you but a pleasant memory; may that memory linger on, down "into the still evening of your lives."

